# Research Hotspots and Frontiers in Post Stroke Pain: A Bibliometric Analysis Study

**DOI:** 10.3389/fnmol.2022.905679

**Published:** 2022-05-13

**Authors:** Chong Li, Xiaoyi Shu, Xiangyun Liu

**Affiliations:** ^1^School of Kinesiology, Shanghai University of Sport, Shanghai, China; ^2^Shanghai Key Laboratory of Sports Ability Support and Development, Shanghai, China; ^3^Shanghai Frontiers Science Research Base of Exercise and Metabolic Health, Shanghai, China

**Keywords:** pain, stroke, CiteSpace, visual analysis, bibliometric

## Abstract

**Background:**

Pain is a common complication after stroke with a high incidence and mortality rate. Many studies in the field of pain after stroke have been published in various journals. However, bibliometric analysis in the domain of pain after stroke is still lacking. This study aimed to deliver a visual analysis to analyze the global trends in research on the comorbidity of pain after stroke in the last 12 years.

**Methods:**

The publications from the Web of Science (WoS) in the last 12 years (from 2010 to 2021) were collected and retrieved. CiteSpace software was used to analyze the relationship of publication year with countries, institutions, journals, authors, references, and keywords.

**Results:**

A total of 322 publications were included in the analysis. A continuous but unstable growth in the number of articles published on pain after stroke was observed over the last 12 years. The Peoples' R China (65), Chang Gung University (10), and *Topic in Stroke Rehabilitation* (16) were the country, institution, and journal with the highest number of publications, respectively. Analysis of keywords showed that shoulder pain after stroke and central post-stroke pain were the research development trends and focus in this research field.

**Conclusion:**

This study provides a visual analysis method for the trend and frontiers of pain research after stroke. In the future, large sample, randomized controlled trials are needed to identify the potential treatments and pathophysiology for pain after stroke.

## Introduction

Pain is a common complication of stroke, reported in 10–45.8% of patients with stroke (Yang and Chang, 2021; Zhang et al., 2021a). Pain after stroke can hinder the progress of rehabilitation and decrease the quality of life in stroke survivors (Payton and Soundy, 2020; Wang and Liu, 2021; Zhang et al., 2021b). However, due to cognitive impairment or lack of communication, pain after stroke is frequently overlooked. Pain is often missed clinically due to a low disclosure rate. The main subtypes of pain after stroke include central post-stroke pain (CPSP), complex regional pain syndrome (CRPS), shoulder pain, and spasticity-related pain (Delpont et al., 2018; Torres-Parada et al., 2020; Yang and Chang, 2021). Many patients persistently experience at least one subtype. Pain after stroke is not always responsive to conventional painkillers (Choi et al., 2021; Haslam et al., 2021). In addition, owing to unclear pathophysiology, effective methods for the treatment of pain after stroke are still limited.

Given the high incidence of post-stroke pain, an increasing number of researchers have studied pain after stroke, and related articles have been published in academic journals (Elias et al., 2020; Scuteri et al., 2020; Chiu et al., 2021; Zhang et al., 2021b). Some studies have investigated non-drug interventions to relieve pain after stroke (Kadono et al., 2021; Malfitano et al., 2021; Zhao et al., 2021). However, a quantitative analysis of publications on pain after stroke has not yet been conducted.

Data visualization is a technology that uses computer graphics and image processing technology to convert data into graphics and display them on the screen and process them interactively (Chen, 2004). Based on co-citation analysis theory and pathfinding network algorithm, CiteSpace software can analyze literature of specific disciplines or fields from multiple perspectives and draw visual maps to explore the critical paths, research hotspots, and frontiers of the evolution of this discipline or field (Chen and Song, 2019). In recent years, using CiteSpace software combined with relevant authoritative databases to visually analyze the literature of a certain discipline or field has become a hot research topic for scholars all over the world (Yin et al., 2020, 2021; Wang et al., 2021; Wu et al., 2021; Yuan et al., 2021).

To address the weakness of quantitative analysis for studies involved in pain after stroke, the objective of this study is to perform bibliometric analysis for the global scientific research on pain after stroke using CiteSpace. The results of the present study would provide valuable reference information for researchers and promote cooperation among various countries and institutions.

## Methods and Materials

### Data Source and Search Strategy

A bibliometric literature search was performed from 01 January 2010 to 31 December 2021 on the core collection database of Web of Science (WoS). For the search, two Medical Subject Heading (MeSH) terms were used. Term A was “stroke.” Term B was “pain.” The search terms were as follows: TS = (stroke) AND TS = (pain).

### Inclusion and Exclusion Criteria

**Figure 1:**
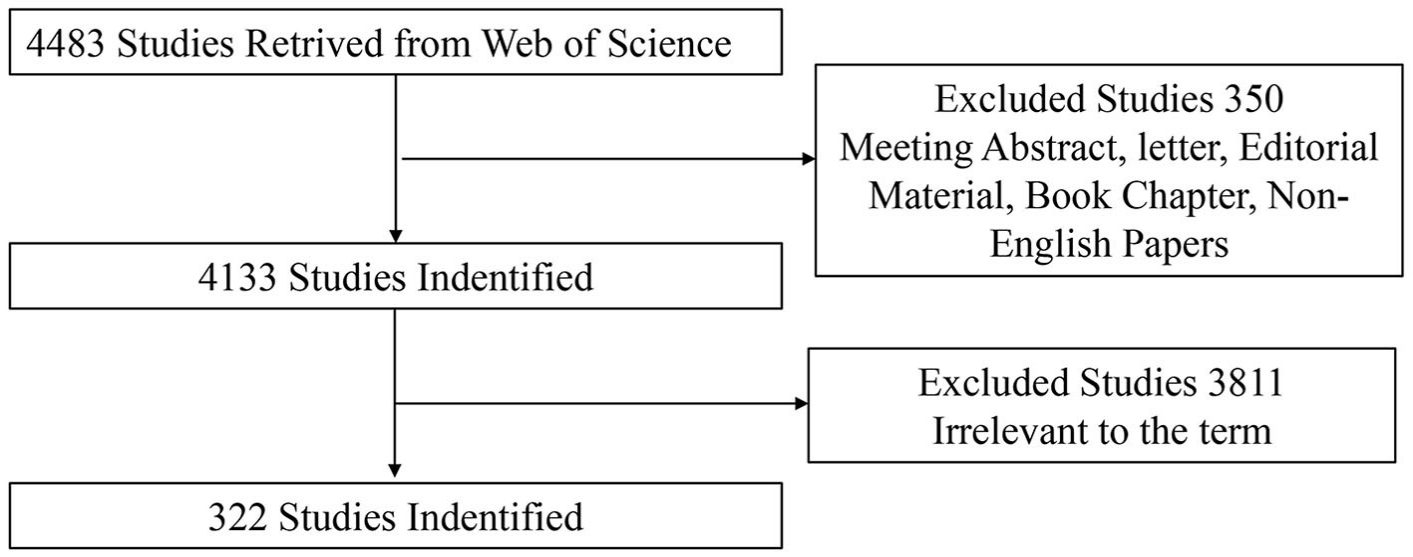
Flowchart of studies inclusion.

Studies related to pain after stroke were selected after screening the title and abstract. Only articles and reviews were included. Other document types, such as letters, commentaries, and meeting abstracts, were excluded. In addition, the publication language was limited to English. The flow chart of the inclusion is shown in Figure 1. Finally, 322 records (276 articles and 46 reviews) were identified for final analysis.

### Analytic Methods

#### Software Parameter Settings

CiteSpace is a visualization software developed by Professor Chen Chaomei (Drexel University, USA) for bibliometric analysis. We used CiteSpace 5.8.R3 to analyze the final records. The ‘Time Sliding’ value was set to 1 year. The type of node was selected according to the purpose of the analysis.

#### Interpretation of Main Parameters in Visualization Map

Cluster view and burst detection: cluster view is carried out on the generated map, and each cluster is labeled by citing the title, keywords, and subject headings in the abstract of the citing reference. The function of burst detection is to detect the situation where there is a great change in the number of citations in a certain period. Thus, it can be used to find the decline or rise of keywords.

Dual-map overlaps: dual-map overlaps are a new method to display the distribution and citation trajectory of articles in various disciplines. As a result, there is a distribution of citing journals on the left side and a distribution of cited journals on the right side. The curve is the citation line, which completely shows the context of the citation.

## Results

### Publication Outputs Analysis

**Figure 2:**
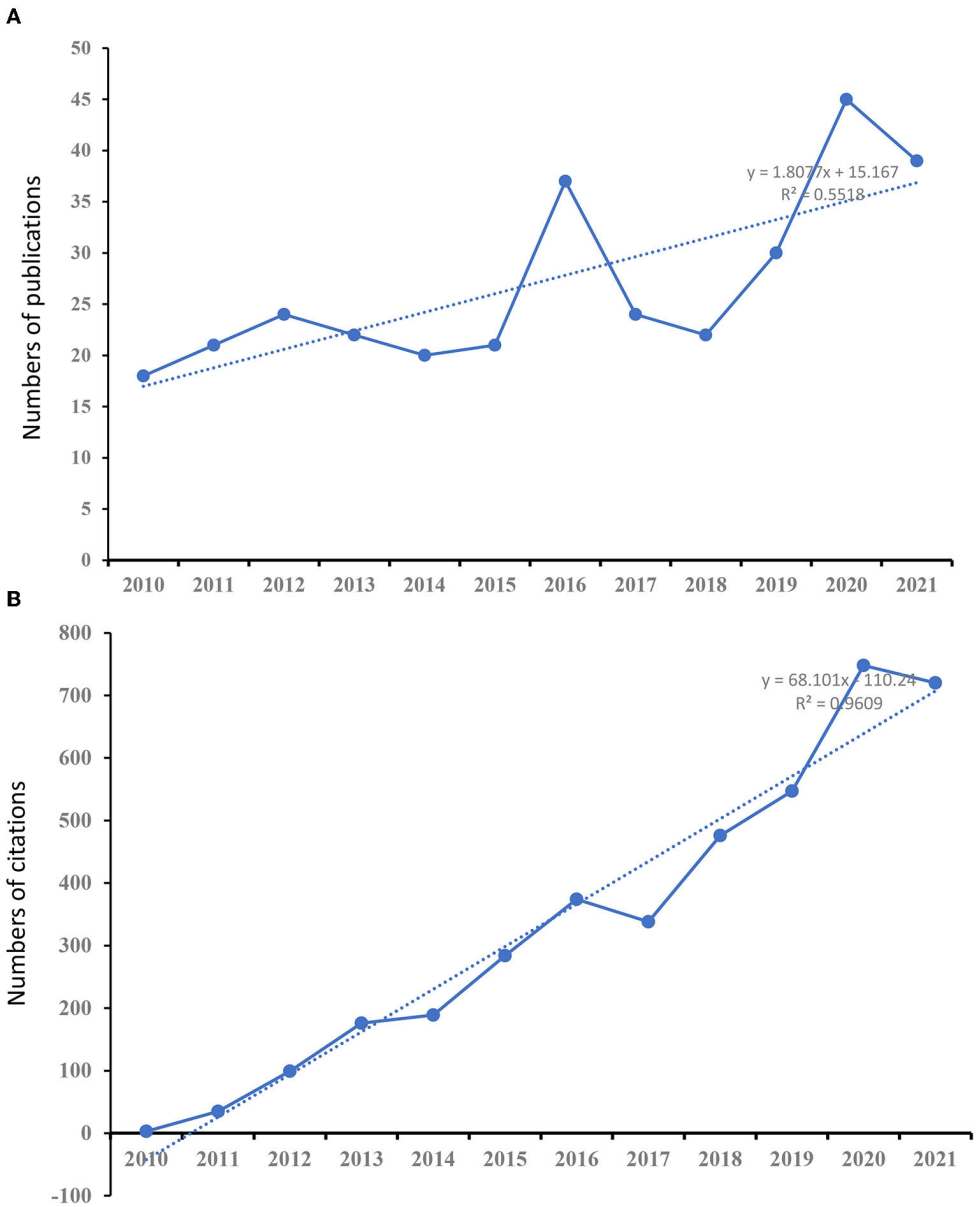
The number of publications and citations. **(A)** The number of annual publications on studies of pain after stroke from 2010 to 2021. **(B)** The number of annual citations on studies of pain after stroke research from 2010 to 2021.

**Figure 3:**
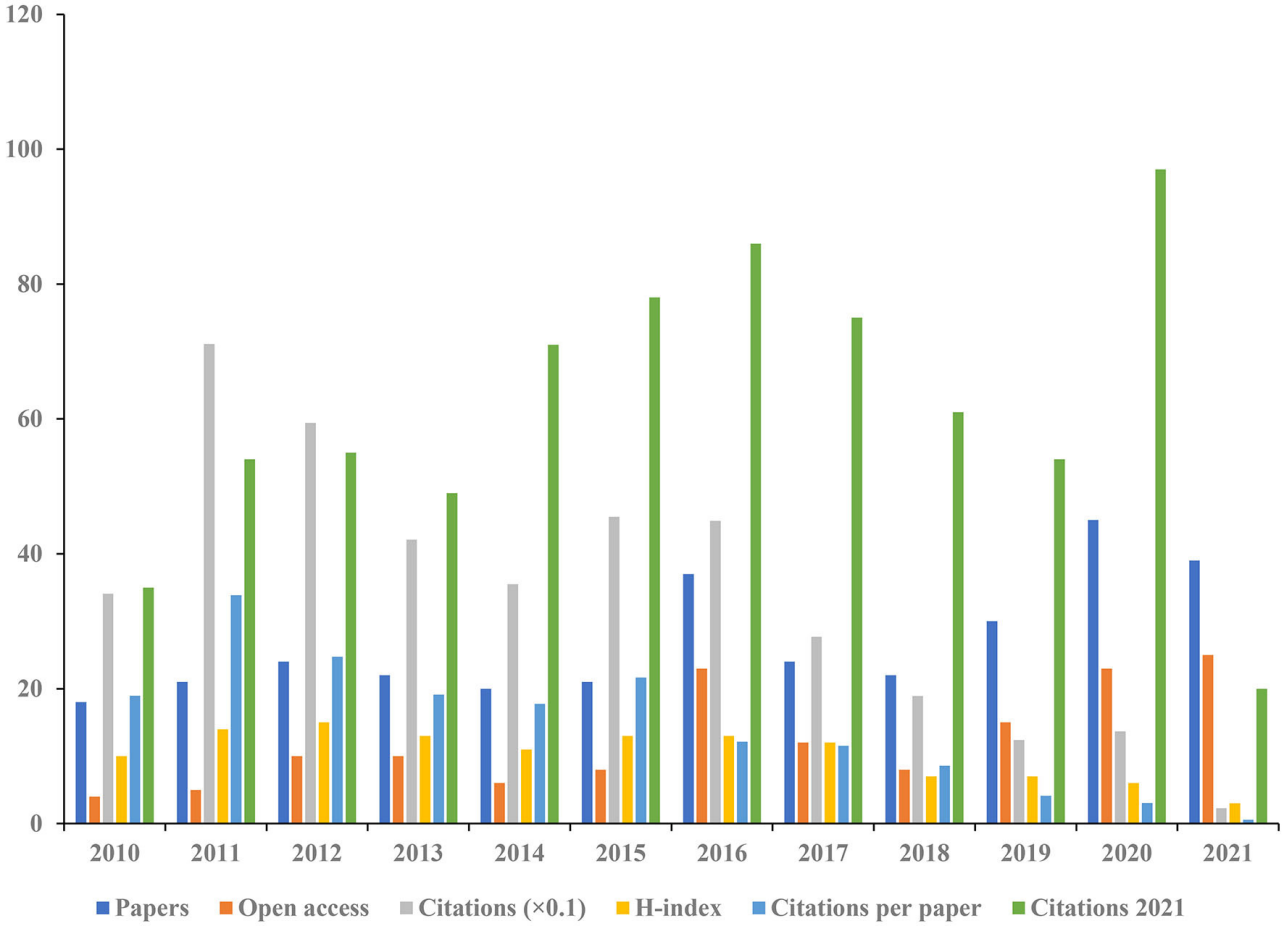
The number of articles, open-access articles, citations, citations per article, citations in 2021, and H-index for each year.

A total of 322 articles were included in the analysis. Figure 2A shows the distribution of annual publication of pain after stroke from 2010 to 2021. The overall trend of publications is positively increasing, and the time trend of publications indicated a significant correlation (*R*^2^ = 0.5518, *p* < 0.01) between the annual publication outputs and the years in the last 12 years. Figure 2B shows the distribution of annual citations of included studies. The number of citations increased from 3 in 2010 to 720 in 2021. The overall trend is positive and the time trend of citations indicated a significant correlation (*R*^2^ = 0.9609, *p* < 0.001). The highest average number of citations per article (33.83), citations (711) occurred in 2011. The highest H-index was found in 2012, and the most published articles (45) were recorded in 2020 (Figure 3).

### Authoritative Journals Analysis

The included 322 studies were published in 156 academic journals. The information of the top 15 journals is shown in Table 1. In the top 15 journals, *Topic in Stroke Rehabilitation* contributed the most published articles (16), followed by *Pain* (12) and *Frontiers in Neurology* (9). *Pain* showed the most citations (335). *Stroke* had the highest average per article citations (45.8) and impact factor (IF = 7.914). *Frontiers in Neurology* presented with the greatest open access value of 9.

**Table 1:** The top 15 journals of articles in pain after stroke.

**Journals**	**Papers**	**Citations (WOS)**	**Citations per Paper**	**Open access**	**WoS Categories**	**IF (2021)**	**Quartile**	***H*-index**
European Journal of Neurology	4	73	18.25	0	Clinical neurology; neurosciences	6.089	Q2; Q2	3
Stroke	5	229	45.8	5	Clinical neurology; peripheral vascular disease	7.914	Q1; Q1	5
Journal of Stroke Cerebrovascular Diseases	5	16	3.2	1	Neurosciences; peripheral vascular disease	2.136	Q4; Q4	3
Clinical Rehabilitation	5	71	14.2	2	Rehabilitation	3.477	Q2	4
BMC Neurology	5	57	11.4	5	Clinical neurology	2.474	Q4	3
PM R	6	65	10.83	3	Rehabilitation; sport sciences	2.298	Q4; Q4	3
Journal of Pain Research	6	47	7.83	6	Clinical neurology	3.133	Q4	4
Journal of Rehabilitation Medicine	7	134	19.14	7	Rehabilitation; sport sciences	2.912	Q3; Q3	5
Archives of Physical Medicine and Rehabilitation	7	180	25.71	1	Rehabilitation; sport sciences	3.966	Q1; Q2	7
American Journal of Physical Medicine Rehabilitation	7	224	32	2	Rehabilitation; sport sciences	2.159	Q4; Q4	7
Medicine	8	47	5.88	8	Medicine	1.889	Q4	4
Journal of Physical Therapy Science	8	58	7.25	7	Sport sciences	\	\	5
Frontiers in Neurology	9	49	5.44	9	Clinical Neurology; Neurosciences	4.003	Q3; Q3	4
Pain	12	335	27.92	1	Anesthesiology; Clinical Neurology	6.961	Q2; Q2	8
Topic in Stroke Rehabilitation	16	199	12.44	1	Rehabilitation	2.119	Q4	9

**Figure 4:**
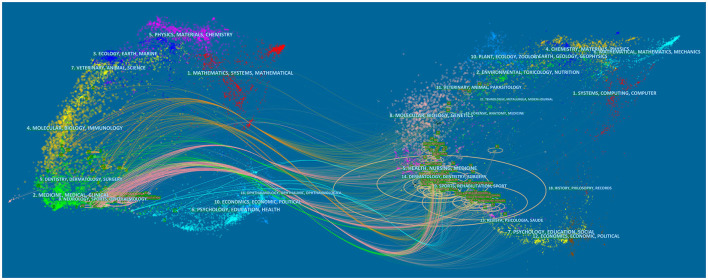
Visualization of dual-map overlays of citing journals and cited journals of 322 studies published from 2010 to 2021. The colored curve indicates the path of citation, which originates from 11 fields of the citing journals on the left and points to 14 fields of the cited journals on the right.

Based on the Blondel algorithm, dual-map overlaps of journals are displayed in Figure 4. The citing journals of 322 studies were mainly from the fields of Medicine, Medical, Neurology, and Sports. The cited journals were mainly from the fields of Health, Medicine, Sports, and Rehabilitation.

### Subject Categories of WoS Analysis

**Figure 5:**
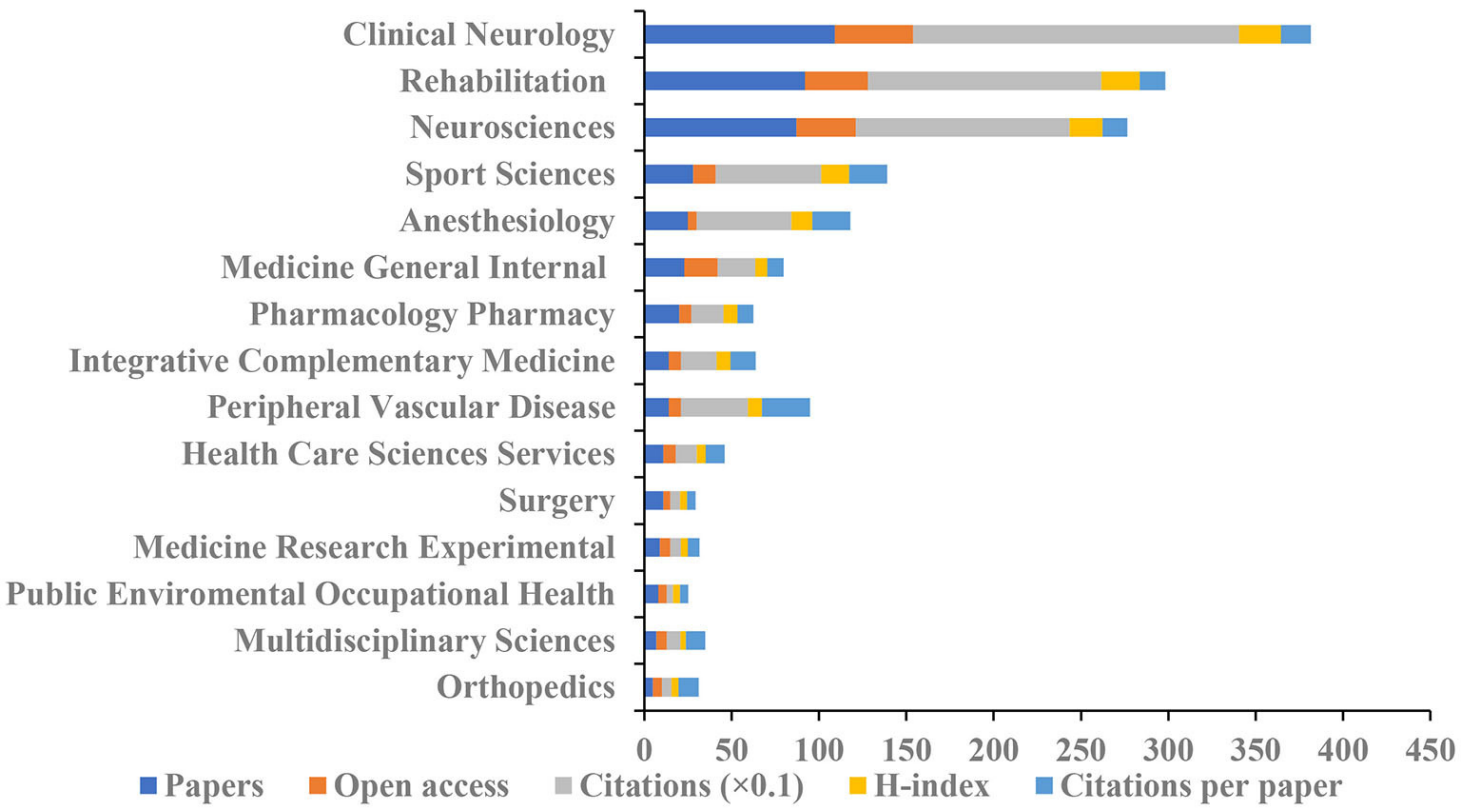
The number of articles, open-access articles, citations, H-index, and citations per article of the top 15 subject categories of Web of Science (WoS).

We classified the 322 papers into 45 subject categories of WoS. The top 15 categories are demonstrated in Figure 5. Among the top 15 subject categories, *Clinical Neurology* ranked the largest number of articles (109), open-access articles (45), citations (1864), and the highest H-index value (24). *Peripheral Vascular Disease* had the largest number of citations per article (27.43).

### References Analysis

**Figure 6:**
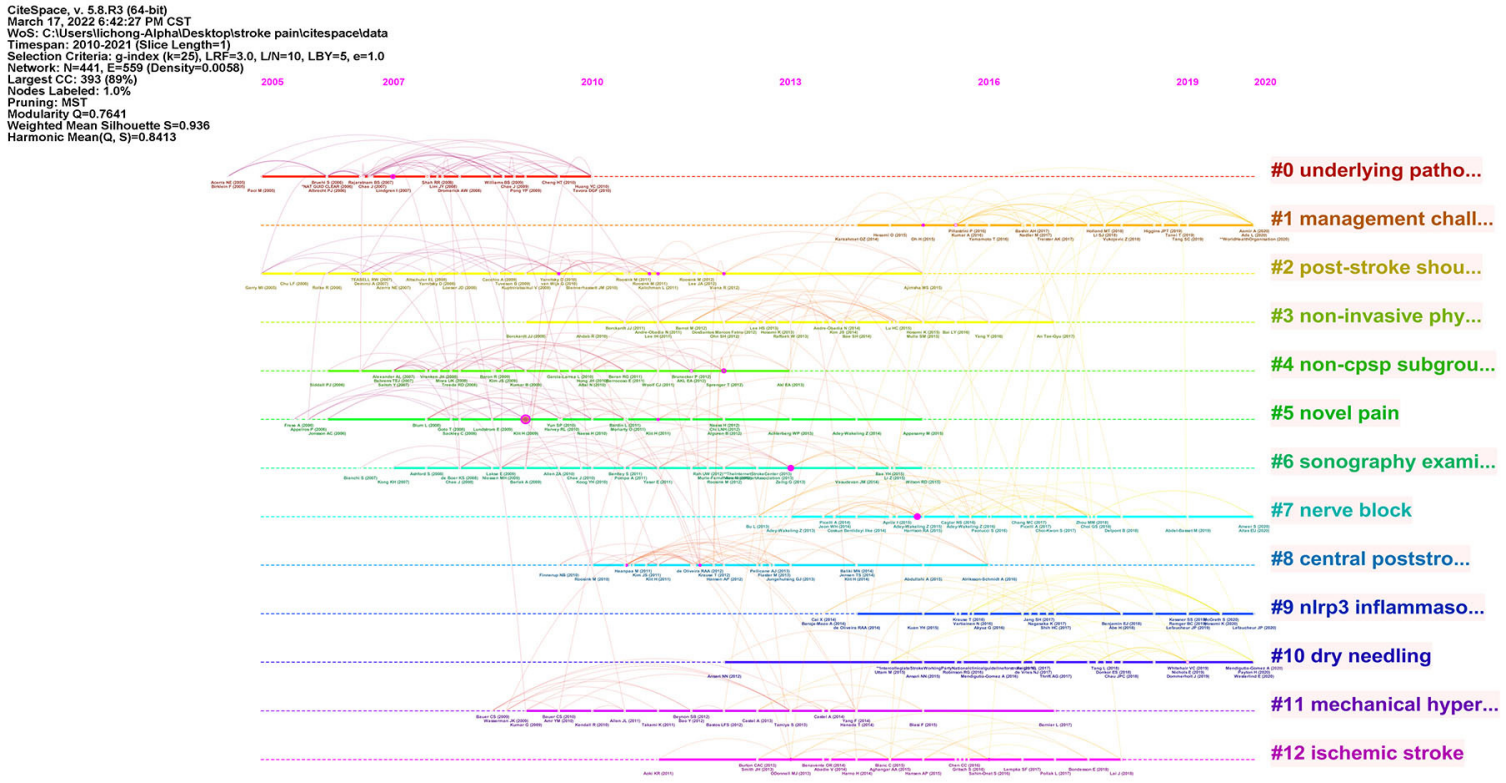
A timeline view of reference co-citation analysis.

The clustered research categories of reference co-citation analysis were divided into 13 groups (#0–12). The timeline view of clusters is shown in Figure 6, which presents the characteristics of the time-span citation information for the cluster domains. The largest cluster (#0) has 42 members, which is labeled as *hemiplegic shoulder pain* by Latent Semantic Indexing (LSI). The most relevant citer to the cluster is “Persistent shoulder pain in the first 6 months after stroke: results of a prospective cohort study” (Roosink et al., 2011). The second-largest cluster (#1) has 34 members labeled as *central post-stroke pain* by LSI. The most relevant citer to the cluster is “Prevalence and management challenges in central post-stroke neuropathic pain: a systematic review and meta-analysis” (Liampas et al., 2020).

The Sigma value can be used to identify innovative references. Five innovative references are summarized in Table 2. Three articles are observational studies and two articles are reviews.

**Table 2:** Five innovative studies about the pain after stroke research among the cited references of the included 322 studies.

**Study**	Sigma^*^	**Journal**	**Study type**	**Sample**	**Intervention**	**Outcomes**	**Highlights**
Adey-Wakeling et al., 2015	0.24	Archives of physical medicine and rehabilitation	A prospective population-based study	301	NA	Subjective reports of onset, severity, and aggravating factors for pain and 3 passive range-of-motion measures	The frequency of poststroke shoulder pain is almost 30%.
Zeilig et al., 2013	0.21	Pain	An observational study	30	NA	The thresholds of warmth, cold, heat-pain, touch, and graphesthesia	The more prominent sensory alterations in the shoulder region suggest that neuropathic factors play a role in hemiplegic shoulder pain.
Sprenger et al., 2012	0.17	Brain	An observational study	10	NA	Magnetic resonance imaging	The ventral posterior nucleus-pulvinar border zone is crucial in the pathogenesis of thalamic pain.
Kalichman and Ratmansky, 2011	0.17	American journal of physical medicine and rehabilitation	Review	NA	NA	NA	The authors categorized the possible underlying pathologies of hemiplegic shoulder pain into three categories: (1) impaired motor control (muscle tonus changes), (2) soft-tissue lesions, and (3) altered peripheral and central nervous activity.
Oh and Seo, 2015	0.17	Pain management nursing	Review	NA	NA	NA	Nurses should be knowledgeable of central post-stroke pain (CPSP), provide precise information to patients and their families, and develop effective nursing care plans that improve outcomes and quality of life for patients with CPSP.

* *Sigma = (centrality+1) burstness (burstness on the index) to identify innovative reference*.

### Authoritative Countries and Institutions Analysis

**Figure 7:**
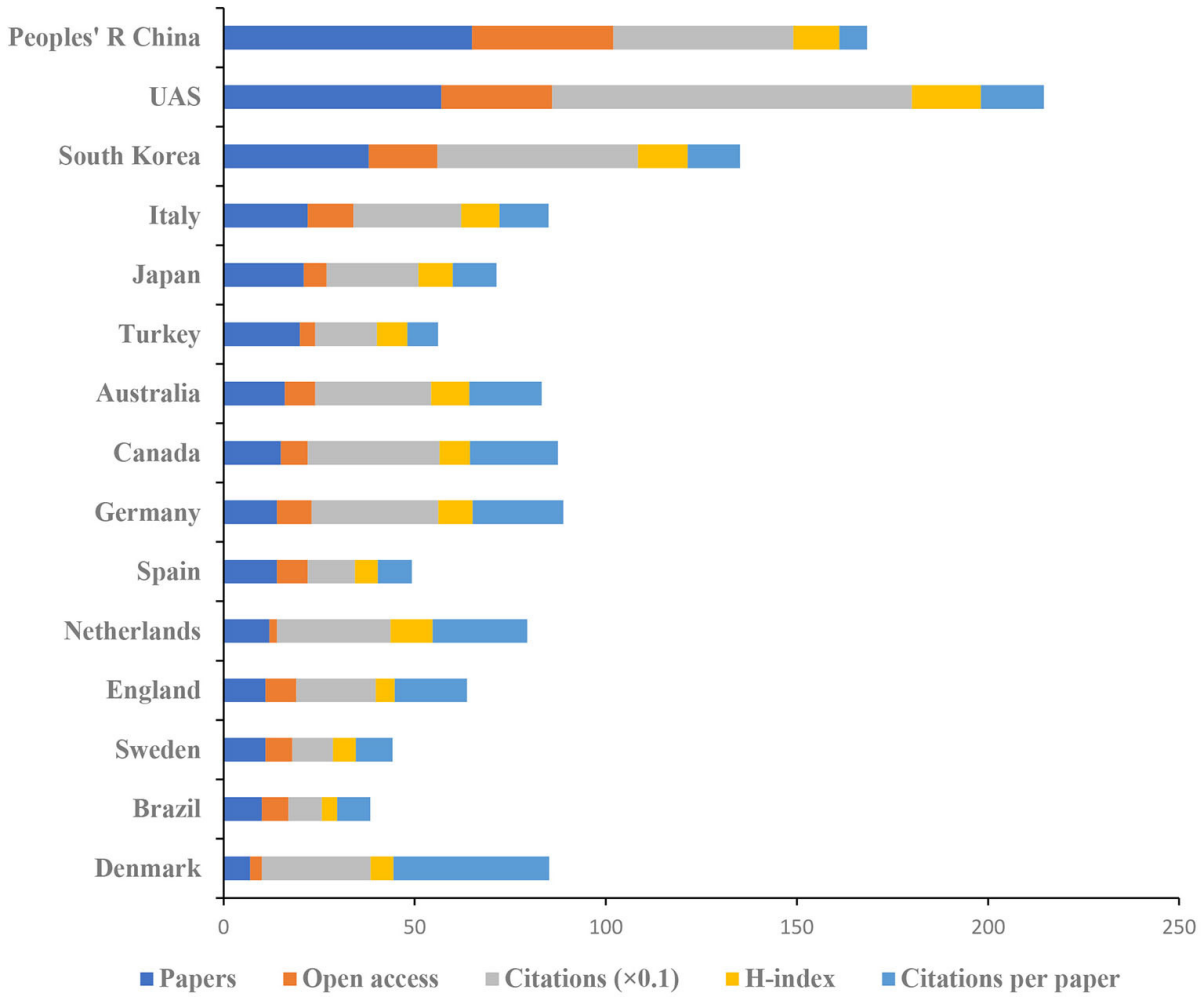
The number of articles, citations, citations per article, and open-access articles, and H-index of the top 15 countries.

Figure 7 shows the top 15 countries in terms of the number of publications on pain after stroke. The highest numbers of publications (65) and open access value (37) were reported in China. The United States had the highest number of citations (941) and H-index (18). Denmark ranked the highest numbers of citation per article (40.71).

**Figure 8:**
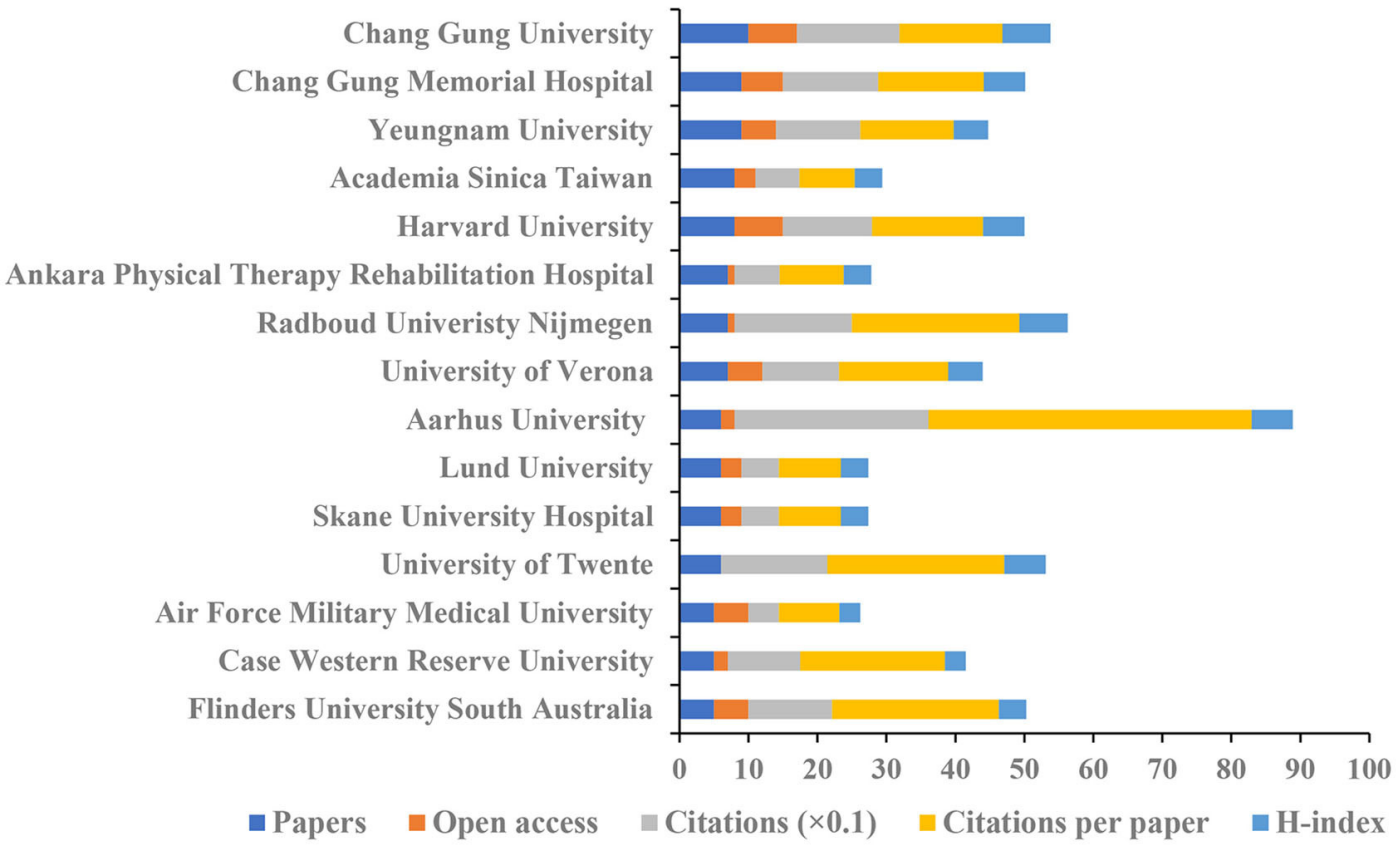
The number of articles, citations, citations per articles, and open-access articles, and H-index of the top 15 institutions.

**Figure 9:**
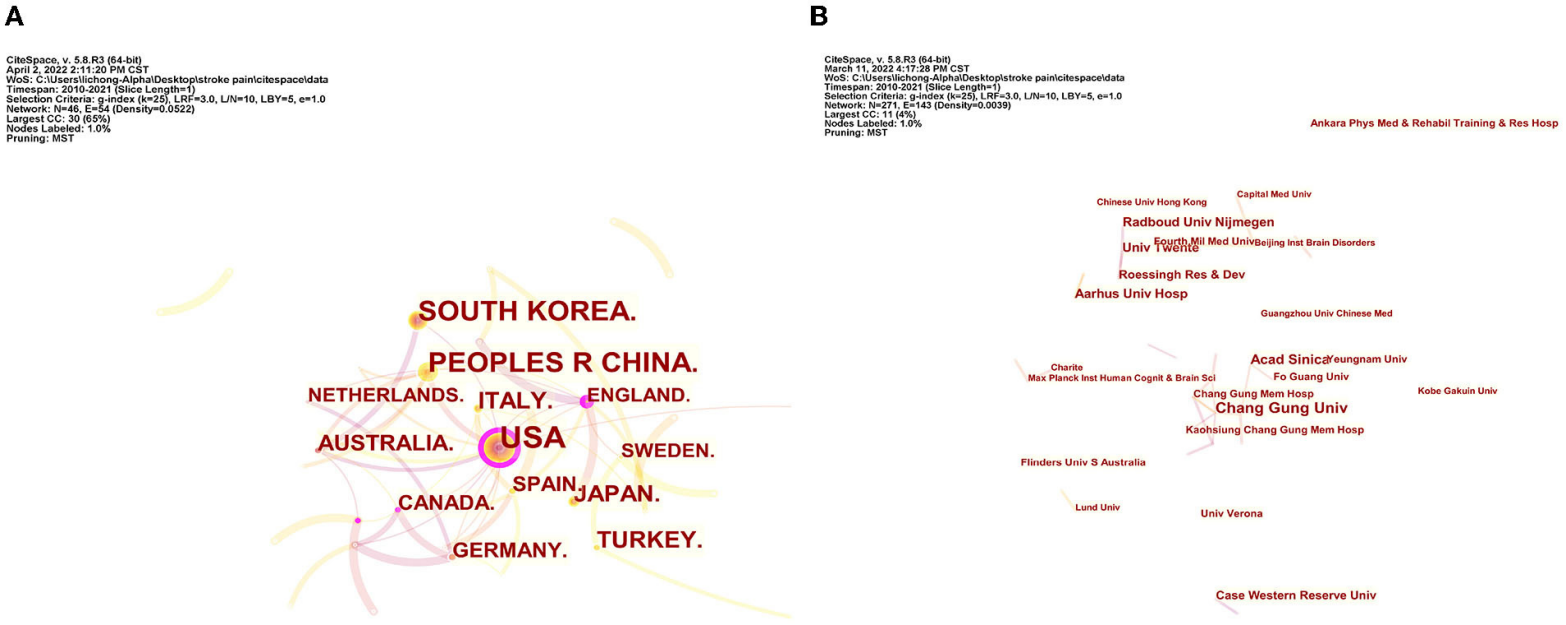
The analysis of countries and institutions. **(A)** Network map of countries engaged in pain after stroke. **(B)** Network map of institutions engaged in pain after stroke.

Figure 8 shows the top 15 institutions based on the number of publications on pain after stroke. The highest amounts of publications (10), open access value (7), and H-index (7) were reported at Chang Gung University. The University of Verona had the highest number of citations (281) and citations per article (46.83). Figure 9 indicates the collaborations between countries and institutions.

### Authoritative Authors Analysis

The 322 papers were contributed by 1,437 authors. The top 15 authors and co-cited authors were ranked in terms of the number of journals published (Table 3). In terms of publications, Shyu BC ranked first (8), followed by Huang YC (7) and Finnerup NB (6). Klit H was co-cited 139 times, followed by Lindgren I (67) and Andersen G (67).

**Table 3:** The top 15 authors, co-cited authors, and co-cited references in pain after stroke.

**Author**	**Papers**	**Co-cited author**	**Cited times**	**Cocited reference**	**Cited times**
Shyu Bc	8	Klit H	139	Klit H, 2009, LANCENT NEUROL, V8, P857, DOI 10.1016/S1474-4422(09)70176-0	37
Huang YC	7	Lindgren I	67	Klit H, 2011, PAIN, V152, P818, DOI 10.1016/j.pain.2010.12.030	21
Finnerup NB	6	Andersen G	67	Sprenger T, 2012, BRAIN, V135, P2536, DOI 10.1093/brain/aws153	20
Huang ACW	6	Bowsher D	64	Lindgren I, 2007, STROKE, V16, P343, DOI 10.1161/01.STR.0000254598.16739.4e	20
ljzerman MJ	6	Kumar B	60	Hansen AP, 2012, EUR J PAIN, V16, P1128, DOI 10.1002/j.1532-2149.2012.00123.x	20
Renzenbrink GJ	6	Kim JS	58	Harrison RA, 2015, CEREBROVASC DIS, V39, P190, DOI 10.1159/000375397	19
Roosink M	6	Jonsson AC	57	Paolucci S, 2016, PAIN MED, V17, P924, DOI 10.1093/pm/pnv019	19
Brogardh C	5	Widar M	55	Kumar B, 2009, ANESTH ANALG, V108, P1645, DOI 10.1213/ane.0b013e31819d644c	17
Fernandez-de-las penas C	5	Chae J	55	Adey-Wakeling Z, 2015, ARCH PHYS MED REHAB, V96, P241, DOI 10.1016/j.apmr.2014.09.007	16
Jang SH	5	Gamble GE	54	Krause T, 2012, J NEUROL NEUROSUR PS, V83, P776, DOI 10.1136/jnnp-2011-301936	14
Jensen TS	5	Vestergaard K	53	Kim JS, 2011, PAIN, V152, P1018, DOI 10.1016/j.pain.2010.12.023	14
Kit H	5	Leijon G	50	Kalichman L, 2011, AM J PHYS MED REHAB, V90, P768, DOI 10.1097/PHM.0b013e318214e976	13
Leong CP	5	Bohannon RW	44	Dromerick AW, 2008, ARCH PHYS MED REHAB, V89, P1589, DOI 10.1016/j.apmr.2007.10.051	13
Lindgren l	5	Roosink M	43	Klit H, 2010, PLOS ONE, V6, P0, DOI 10.1371/journal.pone.0027607	13
Tokuyama S	5	Boivie J	42	Naess H, 2010, J NEUROL, V257, P1446, DOI 10.1007/s00415-010-5539-y	12

### Keywords Analysis

**Figure 10:**
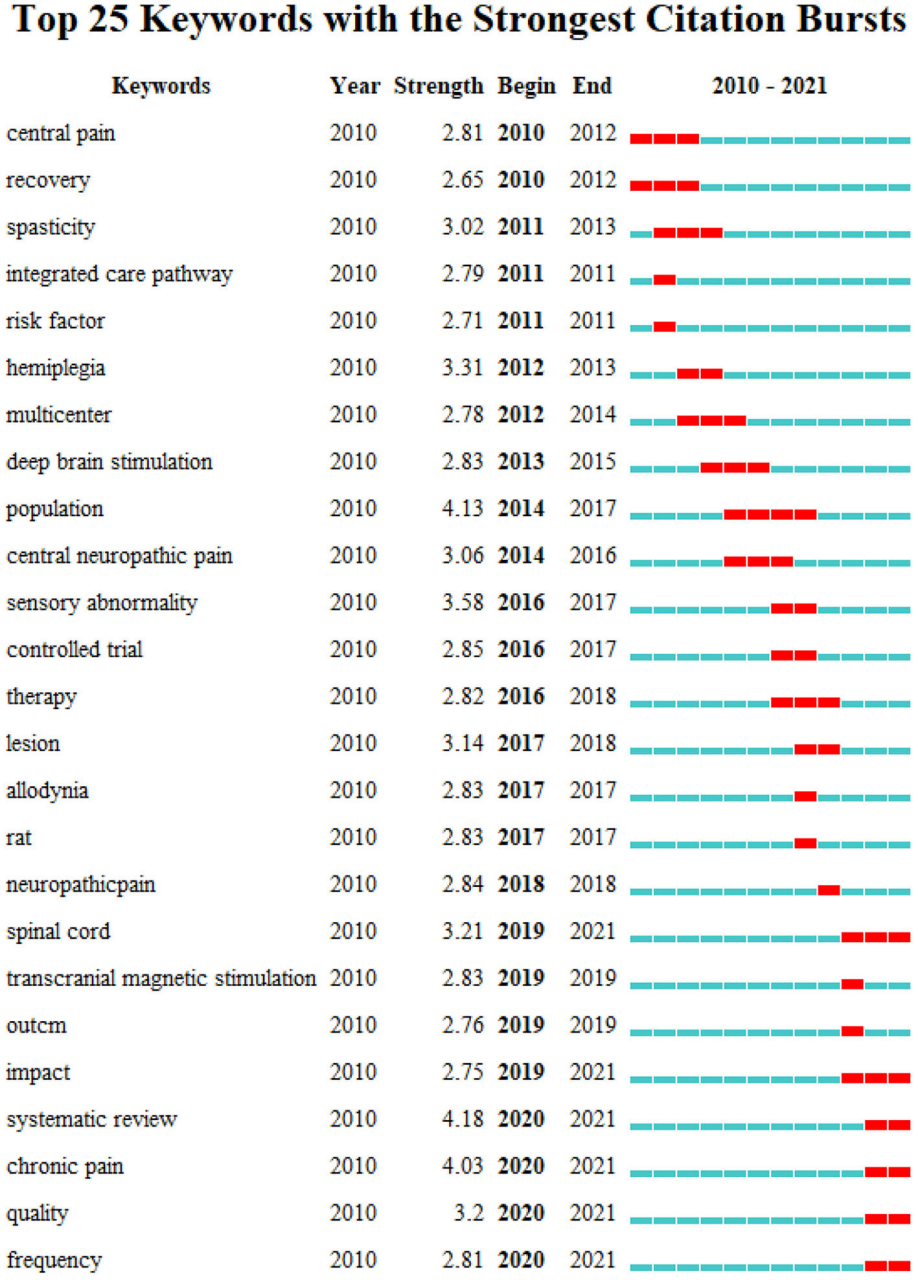
The keywords with the strongest citations bursts of publications on pain after stroke.

The top 25 keywords with the strongest citations bursts from 2010 to 2021 are shown in Figure 10. The strongest citation burst of keywords since 2010 was *central pain*. By the end of 2021, the keywords with the most outbreaks of cited literature included *spinal cord* (2019–2021), *impact* (2019–2021), *chronic pain* (2020–2021), *quality* (2020–2021), *frequency* (2020–2021), and *systematic review* (2020–2021) among the top 25 keywords.

### The Most Frequently Cited Articles Analysis

The top 10 frequently cited articles on pain after stroke are listed in Table 4. The most cited article (95 citations) by Harrison et al. with the title “*Post Stroke Pain: Identification, Assessment, and Therapy*” was published in 2015 in *Cerebrovascular Diseases*. Among the top 10 cited articles, one of them was published in the journals with IF ≥ 10 (*Brain*), five in journals with 5 ≤ IF ≤ 10 (*Stroke* and *Pain*), and four in journals with IF < 5.

**Table 4:** The top 10 articles with the most citation frequency in pain after stroke.

**Title**	**First author**	**Journal**	**Impact factor**	**Year**	**Citations (WoS)**	**WoS categories**	**Category ranking**
Post Stroke Pain: Identification, Assessment, and Therapy	Harrison	Cerebrovascular Diseases	2.762	2015	95	Peripheral vascular disease; clinical neurology	39/65; 133/208
Botulinum Toxin for the Upper Limb After Stroke (BoTULS) Trial Effect on Impairment, Activity Limitation, and Pain	Shaw	Stroke	7.914	2011	89	Peripheral vascular disease; clinical neurology	6/65; 16/208
Central poststroke pain: A population-based study	Klit	Pain	6.961	2011	88	Anesthesiology; clinical neurology; neurosciences	4/33; 23/208; 39/273
Pain following stroke: A prospective study	Hansen	European Journal of Pain	3.931	2012	85	Anesthesiology; clinical neurology; neurosciences	13/33; 75/208; 121/273
Safety and efficacy of pregabalin in patients with central post-stroke pain	Kim	Pain	6.961	2011	75	Anesthesiology; clinical neurology; neurosciences	4/33; 23/208; 39/273
Duloxetine in patients with central neuropathic pain caused by spinal cord injury or stroke: A randomized, double-blind, placebo-controlled trial	Vranken JH	Pain	6.961	2011	71	Anesthesiology; clinical neurology; neurosciences	4/33; 23/208; 39/273
Underlying Pathology and Associated Factors of Hemiplegic Shoulder Pain	Kalichman	American Journal of Physical Medicine & Rehabilitation	2.159	2011	71	Rehabilitation	40/68
Assessing the risk of central post-stroke pain of thalamic origin by lesion mapping	Sprenger	Brain	13.501	2012	68	Clinical neurology; neurosciences	6/208; 10/273
Chronic Pain Syndromes After Ischemic Stroke PRoFESS Trial	O'Donnell	Stroke	7.914	2013	65	Peripheral vascular disease; clinical neurology	6/65; 16/208
Injury of the Spino-Thalamo-Cortical Pathway Is Necessary for Central Post-Stroke Pain	Hong JH	European Neurology	1.710	2010	60	Clinical neurology; neurosciences	179/208; 248/273

## Discussion

### Global Research Trends on Pain After Stroke

This study presents a bibliometric analysis of pain after stroke over the last 12 years. The number of citations has shown a continuous but unstable growth trend yearly, with the most obvious growth trend from 2015 to 2016. The highest amount of published articles (45 publications) was in 2020. The highest numbers of citations and citations per article are in 2011, which are 711 and 33.86, respectively. In addition, the highest H-index (15) value occurred in 2012. These results indicate that studies involved in pain after stroke attracting more and more attention from all over the world.

In terms of authoritative journals, *Topic in Stroke Rehabilitation* (16 publications), *Pain* (12 publications), and *Frontiers in Neurology* (nine publications) contributed the most to the number of published papers. Among the top 15 journals, 13.33% of them were Q1, 20% were Q2, and 13.33% were Q3, indicating the quality of studies on pain after a stroke still needed to strengthen. *Stroke* had the highest number of citations per article (45.8 times). *Pain* had the highest amounts of citations (335 times). The highest H-index value (9) occurred in *Topic in Stroke Rehabilitation*. According to the Journal Citation Reports (2021 edition), none of the top 15 journals had an IF of more than 10. Seven journals had an IF value of 1–3 (*Journal of Stroke Cerebrovascular Diseases, BMC Neurology, PM &R, Journal of Rehabilitation Medicine, American Journal of Physical Medicine Rehabilitation, Medicine*, and *Topic in Stroke Rehabilitation*), four journals had an IF value of 3–5 (*Clinical Rehabilitation, Journal of Pain Research, Archives of Physical Medicine and Rehabilitation*, and *Frontiers in Neurology*), three journals had an IF value of 5–10 (*European Journal of Neurology, Stroke*, and *Pain*). These results indicate that high-quality research should be carried out in the future.

In terms of authoritative countries, China had a dominating contribution to the numbers of published articles (65), followed by the United States (57) and South Korea (38). The top 15 countries are composed of eight European countries, four Asia-Pacific countries, and three American countries. In terms of authoritative institutions, 73% of the top 15 institutions were world-renowned universities. According to international cooperation, the United States and the Chang Gung University had relatively close collaborations with others. Though a wide range of cooperative relationships has been established between various countries and institutions, future studies involved in pain after stroke should focus on international cooperation to carry out multi-center, large sample studies.

### Research Hotspots and Frontiers on Pain After Stroke

According to the subject categories of articles on pain after stroke, *Clinical Neurology* (109) was the most prolific research field, followed by *Rehabilitation* (90) and *Neurosciences* (87). The top 15 subject categories were *Clinical Neurology, Rehabilitation, Neurosciences, Sport Sciences, Anesthesiology, Medicine General Internal, Pharmacology Pharmacy, Integrative Complementary Medicine, Peripheral Vascular Disease, Health Care Sciences Services, Surgery, Medicine Research Experimental, Public Environmental Occupational Health, Multidisciplinary Sciences, and Orthopedics*, indicating pain after stroke is a complex issue that needed multi-disciplinary intervention. *Clinical Neurology* had the highest numbers of open access (45), citations (1864), and H-index value (24).

In terms of reference analysis, the most relevant citer to the largest cluster is “Persistent shoulder pain in the first 6 months after stroke: results of a prospective cohort study.” The authors found that the association of persistent poststroke shoulder pain with restricted, passive, pain-free range of motion, and signs indicative of somatosensory sensitization may implicate a vicious cycle of repetitive trauma that can establish itself rapidly after stroke (Roosink et al., 2011). Therefore, intervention for post-stroke shoulder pain should be focused on maintaining and restoring joint ROM as well as preventing injury and somatosensory sensitization. Future research should investigate different interventions to relieve poststroke shoulder pain.

The evolution of a knowledge domain can be reflected by keywords. Therefore, keyword analysis can reveal emerging trends and provide directions for future research. In terms of count numbers, *shoulder pain* (55) ranked first, followed by *central post-stroke pain* (44). Shoulder pain is one of the most common nociceptive pain syndromes after stroke (Anwer and Alghadir, 2020). The pathophysiology of shoulder pain is thought to be a multifactorial factor, such as glenohumeral subluxation, impingement, rotator cuff tears, glenohumeral capsulitis, and weakness and spasticity are thought to be involved (Precerutti et al., 2010; Chang, 2017). The treatment of shoulder pain after stroke includes pharmacological (antispasmodic medications, Nonsteroidal anti-inflammatory drugs et al.), and nonpharmacological (passive range-of-motion exercises et. al) interventions (Dyer et al., 2020; Souza et al., 2021). In recent years, noninvasive brain stimulation has been proven as a potentially effective intervention to relieve shoulder pain after stroke (Choi and Chang, 2018; de Souza et al., 2019). CPSP is a type of central neuropathic pain. While the pathophysiology of the occurrence of CPSP has not yet been elucidated, some possible mechanisms include hyperexcitability of the spinothalamic tract and central sensitization (Park et al., 2021). Oral medications and non-drug treatments are used to treat CPSP (Ramger et al., 2019; Choi et al., 2021). Recently, a study indicated that prolonged continuous burst stimulation could be a potential treatment for CPSP. Among the top 25 keywords with the strongest citation bursts, *spinal cord* had the strongest strength from 2019 to 2021. A study published in 2020 indicated that perispinal etanercept can provide significant benefits for chronic post-stroke pain (Ralph et al., 2020). Therefore, the current studies on post-stroke pain mainly focus on post-stroke shoulder pain and post-stroke central pain. Future research should focus on well-designed pharmacological and non-pharmacological interventions aiming to relieve pain after stroke. In addition, more studies should focus on the potential pathophysiology of pain after stroke.

### Strengths and Limitations

To the best of our knowledge, this study is the first visual analysis of global trends of pain after stroke based on literature published from 2010 to 2021. In addition, this study contains a comprehensive analysis, such as the number and growth trend of annual publications, different subject sorts of WoS, the relationship among different journals, authors, countries, and institutions, and analysis by different references, citations, and keywords. Nevertheless, this work has some limitations. Because of a limitation of the CiteSpace software, we only analyzed references in the WoS database. Some articles could inevitably have been missed. In addition, large-sample randomized controlled data are lacking.

## Conclusion

In conclusion, this study may help investigators discover the publication patterns and emerging trends of pain after stroke from 2010 to 2021. The most influential author, institutions, journals, and countries were Shyu Bc, Chang Gung University, *Topic in Stroke Rehabilitation*, and the Peoples' R China. The visual map shows the hot research directions of research on pain after stroke in recent years, such as shoulder pain and CPSP. Our bibliometric analysis of 322 studies using CiteSpace software is in line with current clinical studies of research on pain after stroke, indicating that the methodology is valid. In the future, large sample, randomized controlled trials are needed to carry out for pain after stroke.

## Data Availability Statement

The original contributions presented in the study are included in the article/supplementary material, further inquiries can be directed to the corresponding author.

## Author Contributions

LXY contributed to the conception of the study. LC performed the data analyses and wrote the manuscript. SXY and LXY revised the manuscript. All authors contributed to the article and approved the submitted version.

## Conflict of Interest

The authors declare that the research was conducted in the absence of any commercial or financial relationships that could be construed as a potential conflict of interest.

## Publisher's Note

All claims expressed in this article are solely those of the authors and do not necessarily represent those of their affiliated organizations, or those of the publisher, the editors and the reviewers. Any product that may be evaluated in this article, or claim that may be made by its manufacturer, is not guaranteed or endorsed by the publisher.
